# Model of Care for Adolescents and Young Adults with Cancer: The Youth Project in Milan

**DOI:** 10.3389/fped.2016.00088

**Published:** 2016-08-24

**Authors:** Chiara Magni, Laura Veneroni, Matteo Silva, Michela Casanova, Stefano Chiaravalli, Maura Massimino, Carlo Alfredo Clerici, Andrea Ferrari

**Affiliations:** ^1^Pediatric Oncology Unit, Fondazione IRCCS Istituto Nazionale dei Tumori, Milan, Italy; ^2^Department of Hemato-Oncology, University of Milan, Milan, Italy

**Keywords:** adolescent, young adults, model of care, oncology, AYA

## Abstract

Adolescents and young adults (AYA) with cancer form a particular group of patients with unique characteristics, who inhabit a so-called “no man’s land” between pediatric and adult services. In the last 10 years, the scientific oncology community has started to pay attention to these patients, implementing dedicated programs. A standardized model of care directed toward patients in this age range has yet to be developed and neither the pediatric nor the adult oncologic systems perfectly fit these patients’ needs. The Youth Project of the Istituto Nazionale Tumori in Milan, dedicated to AYA with pediatric-type solid tumors, can be seen as a model of care for AYA patients, with its heterogeneous multidisciplinary staff and close cooperation with adult medical oncologists and surgeons. Further progress in the care of AYA cancer patients is still needed to improve their outcomes.

## Introduction

Adolescents and young adults (AYA) with cancer form a particular subset of patients with unique characteristics, from the peculiar cancer distribution to the complex pathway to diagnosis and the difficulties in access to care, from the insufficient awareness that cancer may arise in this age group (among teenagers, families, and physicians) to the particular psychosocial needs (sometimes inadequately addressed) (1–8). Patients in this age group occupy a hazy area in the overlapping worlds of pediatric and adult oncology (4). They are no longer pediatric patients (often exceeding the upper age limit for admission to pediatric units), but they do not fit the bill for the world of adult oncology either (4, 5). They have been described as inhabiting a “no man’s land” (1, 6), and they are often victims of shortcomings in communications and collaborations between different medical services (7, 8).

## Models of Care

In the last 10 years, the scientific community has started to pay increasing attention to this age group. All over the world, AYA-dedicated programs have been founded and implemented at single institutions or on a national scale, variously involving health-care providers, national societies, governments, and charitable institutions. A major issue to address regards the model of care to adopt, as traditional health-care systems have proved ill-equipped to deal appropriately with these patients’ peculiarities.

Pediatric and adult medical oncologists adopt different organizational models, and Table 1 shows the features of two models of care, their particular strengths and weakness, considering various aspects, such as organization and priorities. A specific and standardized model of care for AYA has still not been developed, and neither the pediatric nor the adult oncological models of care are tailored to these patients’ needs (9, 10). It is still a matter of debate whether a new, dedicated model should be set up or whether (and how) one of the existing models could be adjusted to suit this purpose (11).

**Table 1:** **Comparison between pediatric and adult medical oncology models of care**.

Pediatric model of care	Adult medical oncology model of care
“Family-centered” model. Age-appropriate level of involvement in the relationship. Patients do not have a thorough knowledge of their condition, while parents do	“Disease-centered” model. Direct interaction between leading doctors and patients. Patients have an autonomous, active role in the decision-making process
Multidisciplinary team. Involvement of different specialists (oncologists, surgeons, radiotherapists, nutritionists, specialist consultants, as well as nurses, teachers, psychologists, educators, social workers), taking care of all aspects of patients’ and their family’s lives	Units are often cancer-specific, with a far more classical organization. Multidisciplinarity is “pathology-focused” (surgeons, radiotherapists). The participation of non-medical specialists (e.g., psychological, social and educational support) is still rare, even at referral centers
High staff/patient ratio. This enables more time to be dedicated to the single nuclear family; relatively more resources than other units	Lower staff/patient ratio. More limited resources; fewer opportunities for medical staff to spend time on individual patients, which may influence quality of care
Standardized protocols or multicenter clinical trials within national or international multicenter collaborative networks	Fewer chances of patients being enrolled in cooperative clinical trials. Screening, prevention and early diagnosis programs
Phase 3 trials	Phase 1–2 trials, focusing on the search for new treatments

Care for AYA with cancer should ideally be patient-centered, with a view to acknowledging each patient’s level of autonomy and maturity though, dealing with parents or other significant parties involved is still fundamentally important. A key element in developing a dedicated model of care is to promote patients’ normalcy, to enable them to continue living as normally as possible, experience the typical rites of passage of this age, and reach their developmental milestones despite the cancer diagnosis (12).

A solid multidisciplinary team is essential to take care of their complex clinical and psychosocial needs, so not only oncologists, radiotherapists, and surgeons are involved but also nurses, physiotherapists, psychologists, educators, fertility and sexual consultants, teachers, social workers (skilled in labor law, as some patients in this age group will have already finished school and started working), and spiritual assistants (1, 13). A major problem lies in that AYA can be affected by a variety of pathologies; 60–70% of tumors in adolescents are typical “pediatric malignancies,” but the proportion of “adult-type malignancies” increases with age (4, 14–16). That is why the multidisciplinary staff should theoretically include pediatric and adult medical oncologists – or possibly a new professional figure, the AYA oncologist, expert in both fields.

But there are some obstacles to overcome (17).

The close cooperation between pediatric and adult oncologists fundamental to ameliorating AYA patients’ outcomes and quality of care is still hard to achieve. The creation of teams that include both pediatric and adult medical oncologists might appear to be the solution, but it is difficult to put into practice. In attempting to deal with this challenge, there are various issues to consider, such as the fact that the two disciplines often have different working methods, different priorities, different classification, staging, and grading systems (18–20), all of which make it complicated even to compare and share data and competences. Using pediatric protocols to treat AYA patients with pediatric malignancies, regardless of their age, has produced better results in terms of outcome for various tumor types (4, 21–23). On the other hand, pediatric oncologists may not be perfectly skilled in treating malignancies typical of adulthood that might also affect teenagers, and vice versa (6, 16, 17). Previous experiences of developing targeted projects mostly took place at pediatric oncology units, while others were developed as spin-offs of adult services – they rarely arose from direct collaboration between adult and pediatric oncology teams.

It might be worth considering the feasibility of training a new figure, the dedicated “AYA oncologist.” This would entail not only developing specific training programs but also dedicated AYA units, national and international organizations, and cooperative groups. The chances of success of such a proposal are still uncertain; however, such a dedicated health-care provider should master the care of a vast array of different pathologies, which seems unlikely for the time being (17). At the same time, there is little likelihood of new AYA oncology units being created and having different specialists, each of them with a particular AYA focus and expertise. Probably, the most feasible solution is to develop multidisciplinary teams with experts in both AYA care and specific malignancies, who work together with pediatric and medical oncologists (17). The competences needed to work with patients of this age have been analyzed in various works (24, 25). Beyond the expertise in treating adult and pediatric malignancies, they cover various issues, which would demand specific training, on how to give age-appropriate information about the disease, for instance, how to deal effectively with both parents and grown-up patients, as well as team-working and peculiar relational skills (24).

Other key issues include the availability of clinical trials for malignancies concerning this age group and the adequate enrollment of patients in such trials, ongoing research projects, the provision of adequate AYA-dedicated spaces in hospital wards, and the problems of funding AYA programs and measuring their efficacy. The limited access of these patients to referral centers and their inadequate inclusion in clinical trials have probably played a significant part in the limited improvement in their outcomes compared with what has happened in other age groups (4, 7, 15, 26–29). When developing innovative programs, special attention must be paid to economic entailments and institutional support; acceptance as a standard of care is fundamental to the establishment of structured programs. A key requisite in order to receive public investments is to demonstrate the benefit of a program, its affordability or even its capacity to generate revenue, but this may often be far from easy. In some cases, funding may be obtained by redistributing available economic resources, or from peer-reviewed research grants or charitable endowments. Measuring the efficacy of a program is very challenging: providers have to measure their indirect benefits, which might include the development of research projects and collateral services in different fields, such as psychosocial appraisal, fertility preservation programs, and studies on tumor biology, as well as the number of patients enrolled in clinical trials, ongoing research and publications, and patient and provider satisfaction (30).

## The Model of the Youth Project of Milan

Among numerous projects developed in different countries, the Youth Project of the Istituto Nazionale Tumori (INT) stands out in some particular aspects (www.ilprogettogiovani.it). It was founded in 2011 at the Pediatric Oncology Unit of the INT in Milan. Unlike other Italian pediatric oncology departments, which are part of a children’s hospital or a pediatric unit in a general hospital, the INT’s Pediatric Oncology Unit is part of a comprehensive cancer hospital (31). This fact, together with the decision not to consider upper age limits for admitting patients, has enabled a solid cross-cooperation with medical oncologists and surgeons dealing with adult patients, which has generated a great deal of experience of cancer types that trespass age boundaries (32), i.e., adult-type tumors occurring in children (33, 34) and pediatric-type tumors affecting older patients (21, 35). The Youth Project is dedicated to adolescents (15–19 years old) and young adults (up to 25–29 years old) with pediatric-type solid tumors. It was developed as an offshoot of existing activities and involves the same medical and nursing staff at the Pediatric Oncology Unit. It represents a new organizational and cultural approach to these patients’ care that focuses on promoting their normalcy. Other principal founding aims were to optimize clinical aspects regarding AYA (e.g., the enrollment of older patients in clinical trials, psychosocial support, fertility-preserving facilities) and the setup of dedicated spaces and support projects (Table 2). This solution was considered much more feasible than the *de novo* establishment of an independent AYA unit. Within the multidisciplinary team, two doctors, a psychologist, and a youth worker are especially committed to working with patients in this age range. The costs of establishing the Project (dedicated space setup, salaries for the dedicated psychologist and educator, activity planning, and materials) are covered by private funds (Associazione Bianca Garavaglia), without incurring any additional expense for the public administration (31).

**Table 2:** **Peculiar aspects of the Youth Project at the Istituto Nazionale Tumori in Milan**.

Clinical aspects	
Patients’ inclusion in clinical trials	Clinical protocols for all tumor types occurring in AYA are available and enrollment in trials is encouraged
Psychosocial support	Three specialists in clinical psychology are permanent members of the staff; one of them is specifically dedicated to AYA patients; psychologists actively cooperate with the spiritual assistant (a daily presence at the unit), educators (one dedicated to AYA), social workers, and teachers (one high-school teacher)
Fertility-preserving facilities	Sperm cryopreservation and oocyte or ovary tissue cryopreservation for patients considered at risk (in cooperation with gynecology departments at other centers in Milan)
Access to care after completing cancer therapy	Different schemes according to the risk categories; yearly phone contact for “low-risk” patients (i.e., patients who did not receive heavy burdens of therapy – no radiation, no alkylating agents); transition to adult hospital departments for patients needing specific expertise (e.g., patients with melanoma); dedicated programs for “high-risk” patients (e.g., neurological and psychophysical rehabilitation for survivors of brain tumors; cardiological monitoring for patients given anthracyclines; monitoring of endocrine functions; early breast screening program after radiotherapy)

**Age-dedicated spaces**	
Multifunctional room	A 30 m^2^ room for socializing, recreation, and activities, with TV, couches, some computers with Internet connections, musical instruments, a library with books, magazines, and DVDs, an area for listening to the radio, a corner for applying face make-up
Classroom	A 25 m^2^ room, with some computers, where teenagers can go to study (alone, with teachers or classmates) or to read
Gym	A 30 m^2^ gym on the same floor as the ward, equipped with exercise bike, rowing machine, treadmill, and various other equipment; personal trainers with specific skill are there three times a week to assist patients wanting to exercise

**Support projects**	
2012: fashion collection (B.LIVE)	During this 6-month project, 24 patients designed their own fashion collection and organized a fashion show with a well-known fashion designer (Gentucca Bini) acting as art director
2013: song writing (Clouds of Oxygen)	20 patients took part in this 8-month project on the use of music as a form of expression; with help from a famous Italian rock band (Elio e Le Storie Tese), patients wrote and recorded a song called “Clouds of Oxygen”
2015: writing classes	25 patients worked together to write a novel under the leadership of a writer/teacher (Lorenza Ghinelli); the novel tells the story of some superheroes, created by the patients involved; the project lasted 8 months; the novel will be published in the form of a graphic novel
2016: photography project	Together with four professional photographers, 30 patients learned the principles of photography; on completing this 8-month project, they are currently organizing an exhibition entitled: “The Search for Happiness”
Sports project	Indoor sports activities at the gym attached to the ward; outdoor activities (football matches, sailing activities)
Football therapy	In collaboration with the Football Club Internazionale Milano, patients are invited to the stadium for every home match, where they watch the game in a dedicated Sky Box
Facebook	A password-protected group on Facebook gives patients a chance to share their feelings and ideas, and work together on activities and assignments, even if they cannot attend hospital meetings; the educator and dedicated psychologist act as moderators for the group

The support projects include creative activities (fashion collection, song writing, novel writing, a photography course, and exhibition), designed to occupy patients for some months, and other support activities (sports, football matches, Facebook), which are organized differently and over a longer period. Creative activities, in which art is used as the principal means of expression, are run by experts working together with an educator, and this solution has proved very successful in helping patients feel part of a group, cope better with their disease, and feel special. These projects should also be seen as a complement to the medical and psychological activities undertaken to sustain the continuity of patients’ lives. Thanks to these activities, patients find a way to express unvoiced feelings, process their thoughts, and integrate the negative experience of the disease in their personality (12, 36–38). Patients meet once a week and the Youth Project password-protected group on Facebook is used daily to take part in the activities and share ideas, even if patients are at home and cannot come to the hospital. In a recent paper, we published a list of reproducible parameters that may demonstrate the value of dedicated AYA programs. These metrics can be used to assess the effectiveness of our own project: e.g., the number of newly diagnosed adolescent patients accessing our Unit has increased of 25% since the Youth Project foundation; 82% of these patients were included in clinical trials; 59% received fertility preservation measures; 70.6% received psychosocial support; 72% actively participated in support activities (39).

To gain acceptance as a standard of care at community and government level, local programs such as the Youth Project have to be part of a far-reaching project, involving a national program. In 2014, the SIAMO project (Società scientifiche italiane Insieme per gli Adolescenti con Malattie Onco-ematologiche, Italian Scientific Societies Together for Adolescents with Onco-Hematological Diseases, www.ilprogettogiovani.it) was launched, with the support of the national health service and government. The creation of a broader program has been possible thanks to a greater awareness of the needs of AYA within the community, the cooperation of different scientific societies, and also the efforts of INT Youth Project members (40). SIAMO aims to increase public awareness, to define a national comprehensive program to bridge the gap in the quality of care and outcome for these patients, and at the same time to serve as a reference for institutions and mass media to promote AYA-dedicated schemes. Among several initiatives, SIAMO conducted a survey among healthy adolescents to investigate their awareness of health issues and especially cancer, its signs and symptoms. The results showed that, although they are concerned about their own well-being, they often postpone reporting any symptoms they are experiencing to an adult, as other studies had previously reported (41, 42). Enlightened by the effective need to improve awareness, SIAMO launched two campaigns [#fattivedere – Italian term with a double meaning: “Don’t hide!” and “Get a check-up,” and “There is no reason why” (43)] targeted to reduce the diagnostic delay that AYA with cancer experience. Both of these campaigns were linked to the SIAMO website page that provides information on how to interpret symptoms.

A further goal of SIAMO is to include AYA’s needs in Italy’s next National Oncology Plan, identifying tailored criteria and facilities that centers need to have in order to treat AYA with cancer, and to establish a network of AYA-dedicated centers.

In conclusion, we have seen a great increase in the attention dedicated to AYA with cancer in the recent years, but we still have to unravel the knots in order to create linear diagnostic paths, clear collaboration among specialists, adequate structures, unhindered access to clinical trials, and finally improve their outcomes.

## Author Contributions

All authors contributed to the conception/design of the work; they contributed specifically to the interpretation of data, worked on its draft, revised it critically and finally approved it. All authors expressed their agreement to be accountable for all aspects of the work in ensuring that questions related to the accuracy or integrity of any part of the work are appropriately investigated and resolved.

## Conflict of Interest Statement

The authors declare that the research was conducted in the absence of any commercial or financial relationships that could be construed as a potential conflict of interest.

## References

[1] Hollis R Morgan S The adolescent with cancer – at the edge of no-man’s land. Lancet Oncol (2001) 2(1):43–8. 10.1016/S1470-2045(00)00195-9 11905619

[2] Thomas DM Seymour JF O’Brien T Sawyer SM Ashley DM. Adolescent and young adult cancer: a revolution in evolution? Internal Med J (2006) 36(5):302–7. 10.1111/j.1445-5994.2006.01062.x 16650195

[3] Abrams AN Hazen EP Penson RT. Psychosocial issues in adolescents with cancer. Cancer Treat Rev (2007) 33:622–30. 10.1016/j.ctrv.2006.12.006 17434265

[4] Bleyer A Budd T Montello M. Adolescents and young adults with cancer. The scope of the problem and criticality of clinical trials. Cancer (2006) 107:1645–55. 10.1002/cncr.22102 16906507

[5] Ferrari A The challenges of access to care for adolescents with cancer in Italy: national and local pediatric oncology programs. International perspectives on AYAO, part 2. J Adolesc Young Adult Oncol (2013) 3:112–7. 10.1089/jayao.2012.0013 26812189

[6] Ferrari A Bleyer A. Participation of adolescents with cancer in clinical trials. Cancer Treat Rev (2007) 33:603–8. 10.1016/j.ctrv.2006.11.005 17250970

[7] Ferrari A Montello M Budd T Bleyer A. The challenges of clinical trials for adolescents and young adults with cancer. Pediatr Blood Cancer (2008) 50:1101–4. 10.1002/pbc.21459 18360838

[8] Kyle RG Macmillan I Rauchhaus P O’Carroll R Neal RD Forbat L Adolescent Cancer Education (ACE) to increase adolescent and parent cancer awareness and communication: study protocol for a cluster randomised controlled trial. Trials (2013) 14:286. 10.1186/1745-6215-14-286 24011093 PMC3846788

[9] Albritton K Bleyer WA. The management of cancer in the older adolescent. Eur J Cancer (2003) 39(18):2584–99. 10.1016/j.ejca.2003.09.013 14642921

[10] Eden T Challenges of teenage and young-adult oncology. Lancet Oncol (2006) 7(8):612–3. 10.1016/S1470-2045(06)70769-0 16887473

[11] Wilkinson J Young people with cancer: how should their care be organised? Eur J Cancer Care (2003) 12(1):65–70. 10.1046/j.1365-2354.2003.00313.x 12641558

[12] Morgan S Davies S Palmer S Plaster M. Sex, drugs, and rock ‘n’ roll: caring for adolescents and young adults with cancer. J Clin Oncol (2010) 28(32):4825–30. 10.1200/JCO.2009.22.5474 20498401

[13] Proserpio T Ferrari A Veneroni L Giacon B Massimino M Clerici CA Spiritual aspects of care for adolescents with cancer. Tumori (2014) 100(4):130e–5e. 10.1700/1636.17926 25296603

[14] Bleyer WA. Cancer in older adolescents and young adults: epidemiology, diagnosis, treatment, survival and importance of clinical trials. Med Pediatr Oncol (2002) 38(1):1–10. 10.1002/mpo.1257 11835231

[15] Bleyer A Montello M Budd T Saxman S. National survival trends of young adults with sarcoma: lack of progress is associated with lack of clinical trial participation. Cancer (2005) 103(9):1891–7. 10.1002/cncr.20995 15795902

[16] Bleyer A O’Leary M Barr R Ries L Cancer Epidemiology in Older Adolescents and Young Adults 15 to 29 Years of Age, Including SEER Incidence and Survival: 1975–2000. NIH Pub. No. 06-5767:1-14-173-190. Bethesda, MD: National Institutes of Health, National Cancer Institute (2006).

[17] Ferrari A Thomas D Franklin AR Hayes-Lattin BM Mascarin M Van Der Graaf W Starting an adolescent and young adult program: some success stories and some obstacles to overcome. J Clin Oncol (2010) 28(32):4850–7. 10.1200/JCO.2009.23.8097 20479411

[18] Birch JM Marsden HB. A classification scheme for childhood cancer. Int J Cancer (1987) 40(5):620–4. 10.1002/ijc.2910400508 3679589

[19] Fritschi L Coates M McCredie M. Incidence of cancer among New South Wales adolescents: which classification scheme describes adolescent cancers better? Int J Cancer (1995) 60:355–60. 10.1002/ijc.2910600314 7829244

[20] Barr RD Holowaty EJ Birch JM Classification schemes for tumors diagnosed in adolescents and young adults. Cancer (2006) 106(7):1425–30. 10.1002/cncr.21773 16544312

[21] Ferrari A Dileo P Casanova M Bertulli R Meazza C Gandola L Rhabdomyosarcoma in adults. A retrospective analysis of 171 patients treated at a single institution. Cancer (2003) 98(3):571–80. 10.1002/cncr.11550 12879475

[22] Paulussen M Ahrens S Juergens HF Cure rates in Ewing tumor patients aged over 15 years are better in pediatric oncology units. Results of GPOH CESS/EICESS studies. Proc Am Soc Clin Oncol (2003) 22:816a.

[23] Boissel N Auclerc MF Lheritier V Perel Y Thomas X Leblanc T Should adolescents (15-20y) with ALL be treated as old children or young adults? Comparison of the French FRALLE-93 and LALA-94 trials. J Clin Oncol (2003) 21(5):774–80. 10.1200/JCO.2003.02.053 12610173

[24] Gibson F Fern L Whelan J Pearce S Lewis IJ Hobin D A scoping exercise of favourable characteristics of professionals working in teenage and young adult cancer care: ‘thinking outside of the box’. Eur J Cancer Care (2012) 21(3):330–9. 10.1111/j.1365-2354.2011.01322.x 22248098

[25] Hayes-Lattin B Mathews-Bradshaw B Siegel S. Adolescent and young adult oncology training for health professionals: a position statement. J Clin Oncol (2010) 28(32):4858–61. 10.1200/JCO.2010.30.5508 20823410

[26] Burke ME Albritton K Marina N. Challenges in the recruitment of adolescents and young adults to cancer clinical trials. Cancer (2007) 110(11):2385–93. 10.1002/cncr.23060 17918260

[27] Bleyer WA Tejeda H Murphy SB Robison LL Ross JA Pollock BH National cancer clinical trials: children have equal access; adolescents do not. J Adolesc Health (1997) 21(6):366–73. 10.1016/S1054-139X(97)00110-9 9401854

[28] Ferrari A Dama E Pession A Rondelli R Pascucci C Locatelli F Adolescents with cancer in Italy: entry into the national cooperative oncology group AIEOP trials. Eur J Cancer (2009) 45(3):328–34. 10.1016/j.ejca.2008.12.003 19135358

[29] Ferrari A Aricò M Dini G Rondelli R Porta F. Upper age limits for accessing pediatric oncology centers in Italy: a barrier preventing adolescents with cancer from entering national cooperative AIEOP trials. Pediatr Hematol Oncol (2012) 29(1):55–61. 10.3109/08880018.2011.572963 21707221

[30] Taylor RM Fern LA Solanki A Hooker L Carluccio A Pye J Development and validation of the BRIGHTLIGHT Survey, a patient-reported experience measure for young people with cancer. Health Qual Life Outcomes (2015) 13:107. 10.1186/s12955-015-0312-7 26216214 PMC4517652

[31] Ferrari A Clerici CA Casanova M Luksch R Terenziani M Spreafico F The youth project at the Istituto Nazionale Tumori in Milan. Tumori (2012) 98(4):399–407. 10.1700/1146.12631 23052153

[32] Ferrari A Casanova M Collini P Meazza C Luksch R Massimino M Adult-type soft tissue sarcomas in pediatric age: experience at the Istituto Nazionale Tumori in Milan. J Clin Oncol (2005) 23(18):4021–30. 10.1200/JCO.2005.02.053 15767645

[33] Ferrari A Bono A Baldi M Collini P Casanova M Pennacchioli E Does melanoma behave better in younger children than in adults? A retrospective study on 33 cases of childhood melanoma from a single institution. Pediatrics (2005) 115(3):649–54. 10.1542/peds.2004-0471 15741367

[34] Collini P Massimino M Fagundes Leite S Mattavelli F Seregni E Zucchini N Papillary thyroid carcinoma of childhood and adolescence: a 30-year experience at the Istituto Nazionale Tumori in Milan. Pediatr Blood Cancer (2006) 46(3):300–6. 10.1002/pbc.20474 16047353

[35] Spreafico F Massimino M Gandola L Cefalo G Mazza E Landonio G Survival of adults treated for medulloblastoma using paediatric protocols. Eur J Cancer (2005) 41(9):1304–10. 10.1016/j.ejca.2005.02.022 15869875

[36] Ferrari A Veneroni L Clerici CA Casanova M Chiaravalli S Magni C Clouds of Oxygen: adolescents with cancer tell their story in music. J Clin Oncol (2015) 33(2):218–21. 10.1200/JCO.2014.57.9888 25452453

[37] Marris S Morgan S Stark D “Listening to patients”: what is the value of age-appropriate care to teenagers and young adults with cancer? Eur J Cancer Care (2011) 20(2):145–51. 10.1111/j.1365-2354.2010.01186.x 20477855

[38] Veneroni L Clerici CA Proserpio T Magni C Sironi G Chiaravalli S Creating beauty: the experience of a fashion collection prepared by adolescent patients at a pediatric oncology unit. Tumori (2015) 101(6):626–30. 10.5301/tj.5000365 26045110

[39] Ferrari A Silva M Veneroni L Magni C Clerici CA Meazza C Measuring the efficacy of a project for adolescents and young adults with cancer: a study from the Milan Youth Project. Ped Blood Cancer (in press). 10.1002/pbc.26172 27554940

[40] Ferrari A SIAMO: Italian pediatric oncologists and adult medical oncologists join forces for adolescents with cancer. Pediatr Hematol Oncol (2014) 31(6):574–5. 10.3109/08880018.2014.921750 24933414

[41] Veneroni L Mariani L Lo Vullo S Favini F Catania S Vajna de Pava M Symptom interval in pediatric patients with solid tumors: adolescents are at greater risk of late diagnosis. Pediatr Blood Cancer (2013) 60(4):605–10. 10.1002/pbc.24312 23034970

[42] Magni MC Segrè C Martinella VM Finzi C Veneroni L Clerici CA Adolescents’ awareness on health, cancer and tumor prevention. When and why an adolescent decides to consult a physician. Pediatr Blood Cancer (2016) 63(8):1357–61. 10.1002/pbc.25985 27106760

[43] Magni C Maggioni F Ricci A Barisone E Jankovic M Sarlo Postiglione E “There’s no reason why”: a campaign to raise cancer awareness among adolescents. Tumori (2016) 102(3):270–5. 10.5301/tj.5000493 27032701

